# Experimental Model of Rectal Carcinogenesis Induced by N-Methyl-N-Nitrosoguanidine in Mice with Endoscopic Evaluation

**DOI:** 10.7150/ijms.48231

**Published:** 2020-09-10

**Authors:** Vanessa Foresto Machado, Rogerio Serafim Parra, Caio Abner Leite, Stefania Bovo Minto, Thiago Mattar Cunha, Fernando de Queiroz Cunha, Sergio Britto Garcia, Marley Ribeiro Feitosa, Jose Joaquim Ribeiro da Rocha, Omar Feres

**Affiliations:** 1 Department of Surgery and Anatomy, School of Medicine of Ribeirão Preto, University of São Paulo, Brazil.; 2 Department of Pharmacology, School of Medicine of Ribeirão Preto, University of São Paulo, Brazil.; 3 Pathology and Legal Medicine Department, School of Medicine of Ribeirão Preto, University of São Paulo, Brazil.

**Keywords:** colorectal carcinogenesis, low endoscopy, experimental model.

## Abstract

**Background and purpose:** The discovery of chemical substances with carcinogenic properties has allowed the development of several experimental models of colorectal cancer (CRC). Classically, experimental models of CRC in mice have been evaluated through clinical or serial euthanasia. The present study aims to investigate the role of low endoscopy in the analysis of carcinogenesis induced by N‐methyl‐N′‐nitro‐N‐nitrosoguanidine (MNNG).

**Methods:** Thirty C57BL6 mice were divided into two groups: a control group with fifteen animals that underwent rectal instillation of saline solution on day 0 and a carcinogen group with fifteen animals that underwent a 100 mg/kg MNNG rectal instillation on day 0. In both groups, low endoscopies were performed on weeks 4 and 8. We used a validated endoscopic scoring system to evaluate the severity of colitis and colorectal tumor. Euthanasia was carried out at week 12.

**Results:** We observed higher inflammation scores (p <0.001) and a higher number of tumors (p <0.05) in the MNNG group than the control group, both at weeks 4 and 8. A worsening of inflammation scores from the first to the second endoscopy was also noticeable in the MNNG group. There were no bowel perforations related to the procedure, and there was one death in the control group.

**Conclusion:** Low endoscopy in experimental animals allows safe macroscopic evaluation of colorectal carcinogenesis without the need for euthanasia.

## INTRODUCTION

The onset of colorectal cancer (CRC) is a complex and heterogeneous process that has been a field of great interest to researchers 1. For studies related to the development, treatment and prevention of CRC, animal models or cell cultures are used. There are many types of animal models for CRC carcinogenesis, such as those induced by chemical agents, which represent sporadic CRC, instillation of tumor cells into the animals and those induced by genetic modification of animals, which represent hereditary syndromes 2.

An adequate experimental model for the study of carcinogenesis must be one that induces the development of tumors preferentially in the colon, which is a challenge since most carcinogenic agents cause tumors in several places; in addition, the tumor formation process of the model must be reproducible, which can be hampered by problems of a technical nature, since different species of animals, and within them the different lineages, are variably susceptible to the action of different drugs 3.

The discovery of chemical substances with carcinogenic properties has allowed the development of several experimental models of colorectal cancer 4. These compounds, when administered to rodents, produce both benign and malignant colon neoplasms that are similar, in various aspects, to human tumors 5-7.

The use of murine models has advantages such as small size, cost-effectiveness, easy of handling, short gestation time of the animals, anatomical similarities with humans and easy genetic manipulation. For carcinogenesis studies, rapid tumor induction, the possibility of studying the adenoma-carcinoma sequence, and the possibility of using transgenic, knock-out and knock-in animals are other positive points. 7-9.

There is a need for animal models in colorectal cancer research 10. Classically, experimental models of CRC in mice have been evaluated through the health of the animal (body weight, fecal occult blood, and food or water intake) or serial euthanasia. Since 2002, the use of low endoscopy in mice has been reported as a safe method that could allow serial evaluation of the same animal in a period of time as long as periodic mucosal biopsy 11.

The present study aims to investigate the role of low endoscopy in the analysis of carcinogenesis induced by N‐methyl‐N′‐nitro‐N‐nitrosoguanidine (MNNG), a compound with direct chemical carcinogenic action with high affinity and methylation ability for specific regions in the DNA 12. At low concentrations, this agent is able to induce the formation of aberrant crypt foci in the colonic epithelium, which are preneoplastic lesions with high chances of malignant transformation 13.

## METHODS

### Animals

Thirty female C57BL6 mice with an initial age of 8 weeks and weighing approximately 20 g were used. All animals came from the Medical School of Ribeirão Preto - University of São Paulo and were kept in the vivarium of the Department of Surgery and Anatomy under conditions of constant ambient temperature (24°C±1°C), night-day cycle (12:12 h) and relative humidity in air (60-70%). The animals were fed a standard Purina^®^ (Purina, Nestlé, Ribeirão Preto, SP, Brazil) diet and tap water *ad libitum* and accommodated in groups of 5 mice per cage. They were kept under these conditions for a one-week adjustment period. After adaptation, the animals were divided into 2 random groups:

**A. Control group (CONTROL)** - Fifteen animals that underwent rectal instillation of saline solution on day 0. Low endoscopies were performed on weeks 4 and 8. Euthanasia was carried out at week 12.

**B. Carcinogen group (MNNG)** - Fifteen animals underwent a 100 mg/kg MNNG rectal instillation on day 0. Low endoscopies were performed on weeks 4 and 8. Euthanasia was carried out at week 12.

### Anesthesia and carcinogen administration and low endoscopy

All procedures were carried out under inhalation anesthesia with isoflurane (Isoflorano, Cristália Pharmaceutical Chemicals, São Paulo, SP, Brazil) mixed with 100% oxygen (0.5 ml/min flow). After the procedures, the mice were kept on watch and awakened in 3 minutes.

Rectal administration of saline solution or MNNG (Sigma Chemical Co., St Louis, MO, USA) was conducted in the ventral decubitus position, and 100 μL (100 mg/kg) of distilled water-diluted MNNG or saline solution was rectally administered using a number 6 catheter.

The visualization of the rectum was performed with a portable veterinary device (TELE PACK VET x LED, Karl Storz®, Germany), which consisted of an endoscope of 1.9 mm in diameter, light source, camera, monitor and insufflation pump. All exams were recorded for further evaluation. We used to analyze all findings a validated endoscopic scoring system to evaluate the severity of colitis and colorectal tumor 14.

The following findings were considered during endoscopy: the endoscopic inflammation score of Kodani *et al.* 14 and the number of focal lesions (tumors).

The scale takes into account three components:

- Extension and severity of colorectal inflammation (based on perianal findings, wall transparency, mucosal bleeding and focal lesions) on a scale from 0 to 12.
- Quantitative markings of tumor lesions (using graphs and mappings).
- Identification of different types of endoscopic lesions or complications (using decimal identifiers from 0.0 to 0.9).

In addition, from the second decimal place, the number of lesions found per animal was added. For example, if 3 injuries were found it would be added 0.003, if 12 injuries were found it would be 0.012 and so on.

Intestinal irrigation was performed with 0.9% saline solution prior to the procedure. Before scope insertion, the perianal area was evaluated for lesions. After the insertion, it was necessary to inflate air (with a 3 ml syringe) to allow better visualization of intestinal walls. Care was taken not to cause excessive distension or intestinal perforation. The proctoscopy was relatively simple and was performed by the researcher who was skilled in colonoscopy. The mice were small, weighing less than 20 grams, however no accidents were observed during the rectal administration of the carcinogen.

The animals were submitted to medium laparotomy with isolation and removal of the distal colon, which were immersed in 10% formol solution. Processed colons were included in paraffin and submitted to a microtome cut (4µ thickness). Histological analysis was performed and the dysplastic crypts were counted. The animals were submitted to euthanasia by an overdose of Ketamine/Xylazine anesthesia, at week 12.

### Ethics

This project was approved by the Animal Research Ethics Committee of Ribeirão Preto Medical School, University of São Paulo (CETEA-FMRP- USP), Brazil. All animals were kept according to the Committee on Ethics in the Use of Animals.

### Statistical Analysis

A D'Agostinho-Pearson test was used to explore the normality of the distribution. Continuous variables were compared using ANOVA and Bonferroni post hoc test. A *P* value < 0.05 was considered statistically significant. Statistical analysis was performed using GraphPad Prism 5 statistical software (Graph Pad Software Inc., San Diego, California, USA).

## RESULTS

**Figure 1:**
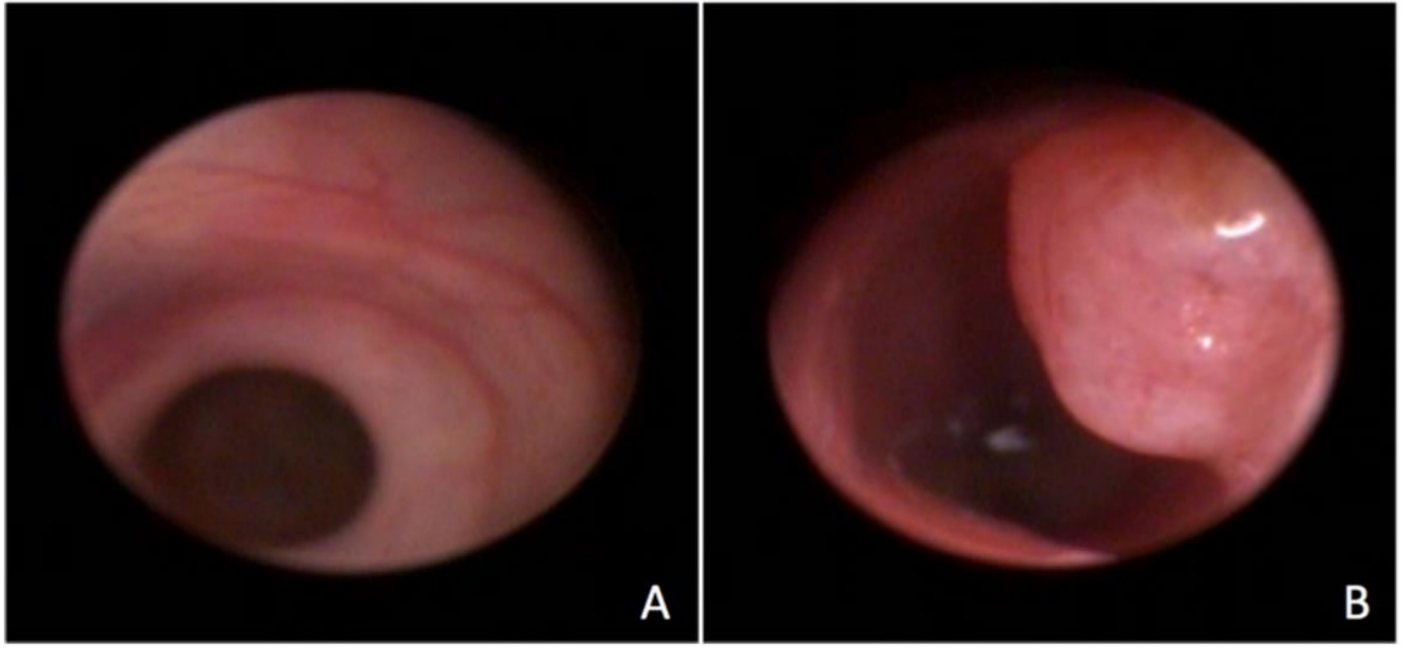
Endoscopic visualization of the normal rectum (A) and of a rectal tumor (B).

The procedure was successfully performed in all animals and allowed visualization of the rectum and part of the colon after a 4 cm progression of the endoscope (figure 1). The mean duration of the procedures was 15 minutes. Although the animals were small (average of 20g) there was no difficulty to introduce the proctoscope and to visualize the mucosa in detail. There were no bowel perforations related to the procedure, and there was one death in the control group 24 h after the procedure attributed to anesthesia complication. The animals remained under observation for 2 hours and no behavioral changes were noted. They were fed normally, remained active and showed no signs of pain. In general, proctoscopy did not interfere with the animals' well-being.

In the control group, there were no signs of inflammation at week 4 (perianal findings, wall transparency or intestinal bleeding). At week 8, changes in wall transparency were observed in 3 animals. In the carcinogenesis group, at week 4, changes in wall transparency were observed in 7 animals, and intestinal bleeding was noted in 5 animals. In the carcinogenesis group at week 8 week, changes in wall transparency were observed in 13 animals, and intestinal bleeding was noted in 7 animals. There was no focal lesion in the control group. In the carcinogenesis group, focal lesions were noted in 6 animals at week 4 and in 9 animals at week 8. The main characteristics of the low endoscopy are described in Table 1.

**Figure 2:**
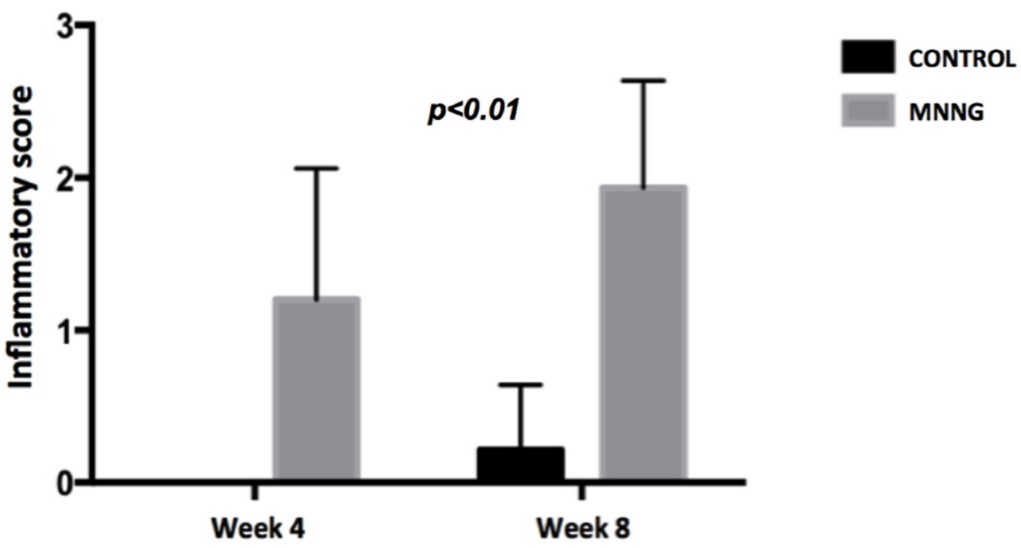
Comparison of mean endoscopic inflammatory scores between groups at weeks 4 and 8.

**Figure 3:**
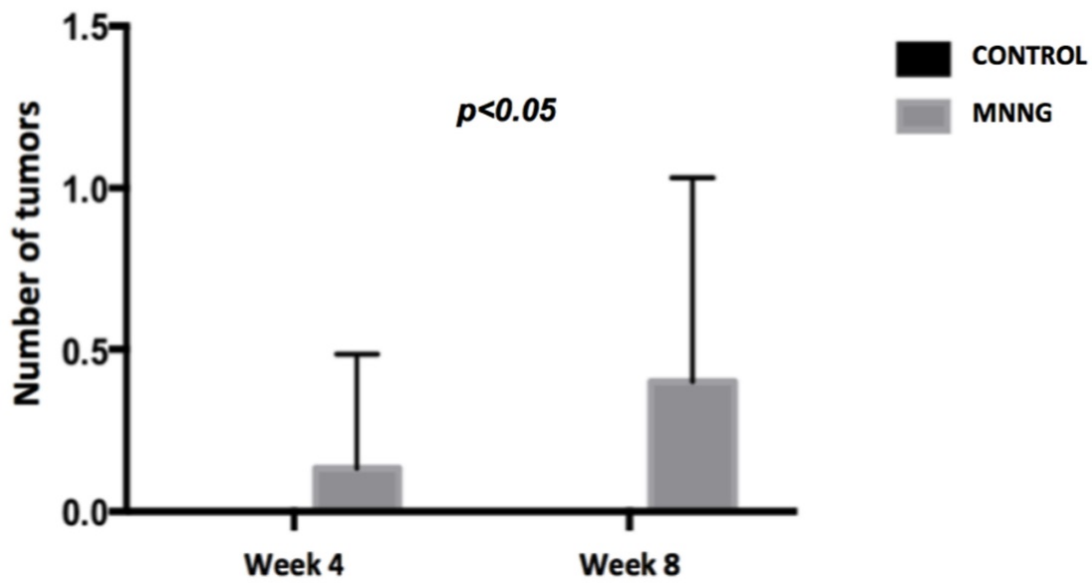
Comparison of the mean number of tumors between groups at weeks 4 and 8.

We observed higher inflammation scores (p <0.001) and a higher number of tumors (p <0.05) in the MNNG group compared to the control group, both at weeks 4 and 8 (figures 2 and 3). A worsening of inflammation scores from the first to the second endoscopy was also noticeable in the MNNG group.

**Figure 4:**
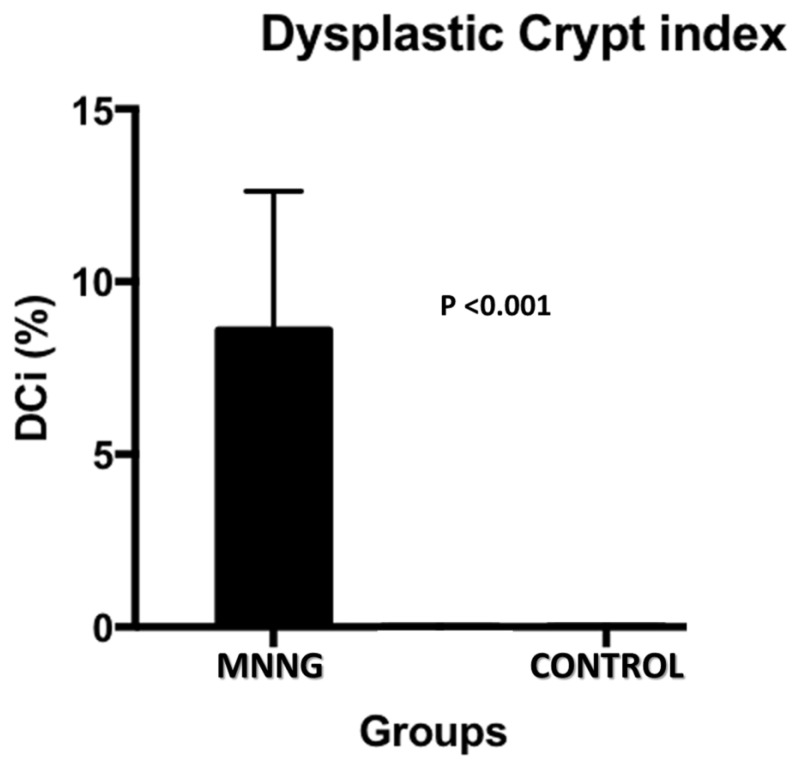
Histological findings of dysplastic crypt in control and MNNG groups.

The histological analysis (dysplastic crypts count) was carried out. The dysplastic crypts index has a direct relationship with carcinogenesis. In the control group, dysplastic crypt was not observed. In the carcinogenesis group the variation of dysplastic crypts ranged from 4 to 16% with an average of 8.6% (figure 4).

## DISCUSSION

In the present study, low endoscopy was used as a method of evaluating the colonic mucosa, as it is an objective exam and the gold standard for screening for colorectal tumors 15. Carcinogenesis was induced with 100mg / kg of MNNG, as described by Maurin *et al.* 16. We observed higher inflammation scores and a higher number of tumors in the MNNG group, both at weeks 4 and 8.

The development and creation of new preclinical models for the study of the CRC carcinogenesis process with the discovery of sensitive and specific biomarkers, not only for initial detection but also to identify patients at risk for disease recurrence or progression despite adjuvant therapy, are critical and essential steps in managing CRC 10.

Low endoscopy in mice has been used as an instrument for evaluating experimental colon and rectal tumors for more than a decade. Using the rigid scope, it was possible to examine the rectum and part of the colon. Classically, for the evaluation of carcinogenesis and the monitoring of tumor progression in animal models, noninvasive clinical parameters, such as weight loss, fluid intake, diet acceptance, diarrhea or hematochezia, are used; these parameters are useful but can present many biases and are not very objective 17. Low endoscopy has the advantage of providing more objective results in relation to tumor or colitis progression, when compared to classical clinical parameters. In addition, low endoscopy allows sequential evaluations in the same animal, being able to measure tumors or to perform serial biopsies, which is not possible when compared to the histological method, in which it is necessary to euthanize the animal 14, 18. Performing serial biopsies in tumors or in experimental models of colitis, there is the possibility of performing cellular and molecular examination, helping to study the adenoma-adenocarcinoma sequence and how to interfere in this process 19.

In addition to biopsies, the working channel can be used to apply substances directly to the tumor or to the inflamed area and evaluate its evolution in treatment compared to the placebo application. Human or murine tumor cells can also be injected into the submucosa for the development of orthotopic tumors in the colon and rectum of the animal. 20.

The greatest difficulty in performing low endoscopy in mice is the small diameter of the colon of these animals; however, the method has already proven to be safe, reproducible and fast. In this study, a total of 60 proctoscopies were performed in 30 mice, with only one death in the first 24 hours after the exam (1.66%). Hensley *et al.* 21 performed 175 exams in 32 mice with 5 deaths (2.9%), 3 during the procedure and 2 in the first 24 hours. The abdominal distension was monitored to prevent distress. The endoscopy was inserted 3cm into de mouse anally and gradually withdrawn over time. Despite being an invasive method, *in vivo* analysis of the progression of colitis and the number and size of tumors in the same animal reduces costs and the number of experimental animals 22.

Endoscopic evaluation is not a substitute for histopathological analysis, but it does provide an alternative for monitoring animal and mucosal lesions in animals still alive; however, when comparing successive biopsies and the endoscopic score in the same animal with colitis over time, an increase in inflammation is noted in both assessment methods 23.

In our country, the use of endoscopic imaging *in vivo* in experimental animals, as well as other means of noninvasive evaluation of the colon of animals, such as ultrasound, nuclear magnetic resonance and computed tomography, although promising, is still rarely used, mainly due to the difficulty of acquiring material by research centers.

In the present study, after applying the MNNG, there was a worsening of the inflammatory score and a progressive increase in the number of tumors over 8 weeks. Similarly, Kodani *et al.* induced colitis with dextran sodium sulfate 3% (DSS 3%) and showed progressive worsening of colorectal inflammation in animals, but without tumor progression 14.

In 2005, Becker *et al.* associated 3% DSS with azoxymethane, and the animals were evaluated on days 0, 20, 40, 60 and 80. Two different scales were used to classify endoscopic exams. The first evaluated the progression of colitis, and the second evaluated the number and size of the tumors. Macroscopic tumors were seen from day 40. Before that, chromoendoscopy was used, which was able to differentiate areas of inflammation from aberrant crypts, that is, premalignant lesions 24.

To our knowledge, our study is the first to evaluate murine carcinogenesis induced by MNNG through digestive endoscopy. It is known that it is a potent topical carcinogen and that its intrarectal instillation in rodents at a dose of 1 to 3 mg/rat/week for 20 weeks induced tumors in 100% of the animals, with 43% adenocarcinomas and 57% adenomas 16. The role of mouse models in colorectal cancer research and the need and importance of orthotopic models were recently described 10. Several models of mouse CRC have been developed over time 2, 5, 24. However, despite their utility as pioneers in the first step of tumor carcinogenesis, these models are extremely limited because they have low tumor development potential; in addition, only a fraction of the mice develop tumors under these conditions, and when tumors develop, they exhibit wide variability in location, diffusion, and differentiation.

Despite being the first to evaluate murine carcinogenesis induced by MNNG through digestive endoscopy, our study has several limitations. First, the mice were small, weighing less than 20 grams. Therefore, in this animal model, there was a need for endoscopic skills both in performing the proctoscopy and in introducing the carcinogen so that there are no accidents or perforation of the rectum. We realized the difficulty of specifying the amount of carcinogen instilled per animal since the animals evacuated after the procedure. Second, we did not perform serial biopsies of the colonic mucosa to compare the endoscopic findings with histopathological findings. However, this model has the ability to follow the various stages of carcinogenesis, allowing safe macroscopic (endoscopic) evaluation from adenoma to the development of cancer, without the need for euthanasia.

## CONCLUSION

In summary, this experimental model with low endoscopy allows safe macroscopic evaluation of colorectal carcinogenesis without the need for euthanasia. The development and creation of new models for the study of the CRC carcinogenesis process are crucial steps in managing CRC.

## Figures and Tables

**Table 1:** Inflammation scores, number of focal lesions and complications observed in low endoscopies on weeks 4 and 8.

		Inflammation score		Focal lesion (n)		Complications	
Animal	Group	Week 4	Week 8	Week 4	Week 8	Week 4	Week 8
1	Control	0	1	0	0	No	No
2	Control	0	0	0	0	No	No
3	Control	0	0	0	0	No	No
4	Control	0	1	0	0	No	No
5	Control	0	0	0	0	No	No
6	Control	0	0	0	0	No	No
7	Control	0	0	0	0	No	No
8	Control	0	0	0	0	No	No
9	Control	0	0	0	0	No	No
10	Control	0	0	0	0	No	No
11	Control	0	0	0	0	No	No
12	Control	0	0	0	0	No	No
13	Control	0	1	0	0	No	No
14	Control	0	0	0	0	No	No
15	Control	0	-	0	-	Death	-
16	MNNG	1	3	0	0	No	No
17	MNNG	2	2	1	1	No	No
18	MNNG	2	3	0	0	No	No
19	MNNG	0	2	0	1	No	No
20	MNNG	2	2	0	0	No	No
21	MNNG	1	1	0	0	No	No
22	MNNG	0	2	0	1	No	No
23	MNNG	3	1	0	0	No	No
24	MNNG	2	3	1	1	No	No
25	MNNG	1	1	0	0	No	No
26	MNNG	0	1	0	0	No	No
27	MNNG	1	2	0	0	No	No
28	MNNG	1	2	0	1	No	No
29	MNNG	1	2	0	0	No	No
30	MNNG	1	2	0	2	No	No
